# Seroprevalence of Antibodies against *Taenia solium* Cysticerci among Refugees Resettled in United States

**DOI:** 10.3201/eid1803.111367

**Published:** 2012-03

**Authors:** Seth E. O’Neal, John M. Townes, Patricia P. Wilkins, John C. Noh, Deborah Lee, Silvia Rodriguez, Hector H. Garcia, William M. Stauffer

**Affiliations:** Oregon Health & Science University, Portland, Oregon, USA (S.E. O’Neal, J.M. Townes);; Centers for Disease Control and Prevention, Atlanta, Georgia, USA (P.P. Wilkins, J.C. Noh, D. Lee, W.M. Stauffer);; Instituto Nacional de Ciencias Neurologicas, Lima, Peru (S. Rodriguez, H.H. Garcia);; Universidad Peruana Cayetano Heredia, Lima (H.H. Garcia);; University of Minnesota, Minneapolis, Minnesota, USA (W.M. Stauffer)

**Keywords:** cysticercosis, neurocysticercosis, Taenia solium, tapeworm, refugees, Bhutan, Myanmar, Burma, Burundi, Laos, Hmong, epidemiology, screening, parasites, cysticerci, United States

## Abstract

Neurocysticercosis (NCC) is a disease caused by central nervous system infection by the larval stage of the pork tapeworm, *Taenia solium*. In developing countries, NCC is a leading cause of adult-onset epilepsy. Case reports of NCC are increasing among refugees resettled to the United States and other nations, but the underlying prevalence among refugee groups is unknown. We tested stored serum samples from the Centers for Disease Control and Prevention Migrant Serum Bank for antibodies against *T. solium* cysts by using the enzyme-linked immunoelectrotransfer blot. Seroprevalence was high among all 4 populations tested: refugees from Burma (23.2%), Lao People’s Democratic Republic (18.3%), Bhutan (22.8%), and Burundi (25.8%). Clinicians caring for refugee populations should suspect NCC in patients with seizure, chronic headache, or unexplained neurologic manifestations. Improved understanding of the prevalence of epilepsy and other associated diseases among refugees could guide recommendations for their evaluation and treatment before, during, and after resettlement.

## Abstract 2

Cysticercosis is an infection caused by a pork tapeworm that creates cysts in different areas of the human body. Sometimes, these parasites can get into the infected patient’s brain and lead to epilepsy or other neurologic disorders. Cysticercosis is most common in developing countries that have poor sanitation and where pigs feed on human waste; however, cases in the United States are increasing. A recent study found that many refugees who settle in the United States, including those from Burma, Laos, Burundi, and Bhutan, have been infected with the tapeworm. The occurrence of cysticercosis among these groups has clinical and public health implications because US physicians might not be familiar with this disease and its symptoms. Cysticercosis should be suspected in refugees who have seizures, headache, or other unexplained neurologic symptoms. Physicians should also be aware that treatment for intestinal parasites, routinely given to refugees before they leave their homeland, can cause serious neurologic reactions in those already infected with the tapeworm.

Cysticercosis is a disease caused by infection with the larval stage of the pork tapeworm, *Taenia solium*. Humans and pigs acquire cysticercosis by ingesting *T. solium* eggs shed in the feces of humans with taeniasis (i.e., infected with an adult intestinal tapeworm). Upon ingestion, tapeworm eggs release oncospheres, which invade the intestinal wall and disseminate through the bloodstream to form cysts throughout the body. The natural lifecycle of *T. solium* tapeworms completes when a human eats pork contaminated by *T. solium* larval cysts because these can then develop into adult egg-producing intestinal tapeworms. This endemic lifecycle occurs primarily in regions where sanitation is poor and where pigs are allowed to roam and access raw human sewage.

Neurocysticercosis (NCC) occurs when cysts develop within the central nervous system (CNS); NCC is the primary cause of illness in *T. solium* infection. The clinical features of NCC cover a diverse range of neurologic manifestations, including seizures, headache, intracranial hypertension, hydrocephalus, encephalitis, stroke, cognitive impairment, and psychiatric disturbances (*1**,**2*). In areas in which *T. solium* infection is endemic, it is a major cause of epilepsy, with 30% of seizure disorder attributable to NCC (*3**–**5*).

Numerous reports document that cysticercosis in the United States occurs primarily among migrants and travelers who are presumed to have acquired their infection in another country (*6**–**9*). Refugees represent a large group of migrants in which the frequency of *T. solium* infection has not been described. Approximately 690,000 refugees resettled in the United States during 2000–2010 (*10*). Resettlement from regions with known pockets of *T. solium* tapeworm endemicity, including Southeast Asia, central Asia, and sub-Saharan Africa, is common. Cysticercosis among resettled refugees has been reported, but the underlying prevalence in refugee populations is unknown (*11**–**15*). Understanding the prevalence of *T. solium* infection could guide recommendations on evaluating and treating refugees before, during, and after resettlement.

During 2010, we used the classic enzyme-linked immunoelectrotransfer blot for lentil-lectin purified glycoprotein (EITB LLGP) to measure the seroprevalence of antibodies against *T. solium* cysts among several refugee populations resettled to the United States in previous years. We present the results, discuss clinical and public health implications, and suggest topics for further research.

## Methods

### Study Populations

**Figure 1:**
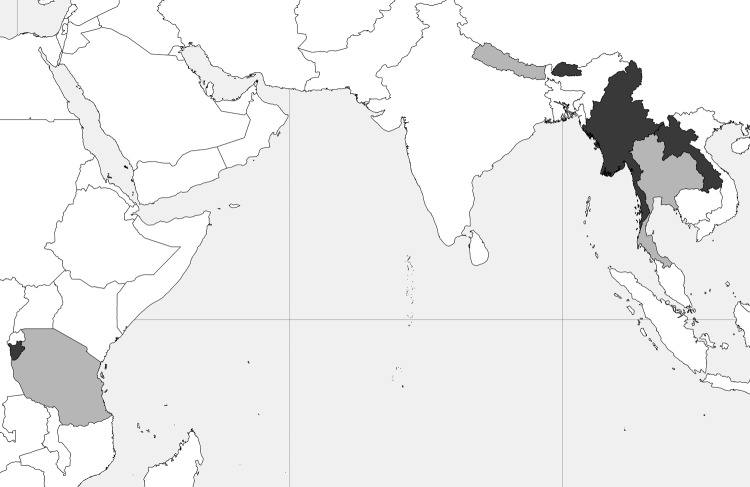
Geographic location and background of refugee populations sampled for antibodies against *Taenia solium* cysticerci by using the classic enzyme-linked immunoelectrotransfer blot for lentil-lectin purified glycoprotein. Countries of origin are shaded dark grey (Burundi, Bhutan, Burma [Myanmar], Laos). Host countries are shaded light grey (Tanzania, Nepal, Thailand). Burundi: ≈14,000 Burundian refugees who lived in camps in Tanzania since 1972 were resettled during 2006–2008. Resettled refugees were primarily ethnic Hutu. Bhutan: ethnic Lhotshampa Bhutanese refugees arrived in Nepal ≈1990. Resettlement began in 2008 and is ongoing, with ≈40,000 resettled thus far. Burma: there has been intermittent influx of refugees into Thailand from Burma since 1984. Resettlement began in 2004 and is ongoing, with ≈90,000 resettled thus far. Resettled refugees in this group are primarily ethnic Karen and Karenni. Laos: refugees from Laos arrived in Thailand as early as 1975, and many resettled soon thereafter. The most recent round of resettlement from the Wat Tham Krabok camp occurred during 2004–2006 with resettlement of ≈16,000 ethnic Hmong refugees.

Refugees who apply for resettlement to the United States are required to undergo a predeparture medical screening examination that includes collection of a peripheral blood sample from persons >15 years of age. The Migrant Serum Bank, established by the Division of Global Migration and Quarantine at the Centers for Disease Control and Prevention (CDC, Atlanta, GA, USA) in 2002, retains a convenience sample of de-identified serum samples from these examinations. Each sample has associated demographic information, including refugee group, age, birth country, refugee camp, and site and date of specimen collection. At the time of this study, ≈31,000 serum samples were available that represented resettled refugee populations from the Middle East, Southeast Asia, and Africa. We identified refugee populations represented in the Migrant Serum Bank in which cases of human cysticercosis or NCC have been reported in the countries of origin (*16*). We then randomly selected serum samples from each of these identified populations to test by EITB LLGP for antibodies against *T. solium* cysts. Populations with limited numbers of samples were excluded because lack of statistical power could impede prevalence estimations. Our final sample comprised 2,001 serum samples from resettled refugees from Laos, Burma (renamed Myanmar in 1989), Bhutan, and Burundi (Figure 1). The institutional review boards at CDC and at Oregon Health & Science University reviewed and approved this study.

### Laboratory Methods

Individual 100-μL aliquots of each sample were separated at the CDC Central Repository, stored in microtubes, and shipped on dry ice to the CNS Parasitic Diseases Research Unit, Universidad Peruana Cayetano Heredia (Lima, Peru), for processing. Serum samples were analyzed by EITB for the presence of antibodies against *T. solium* cysts (EITB LLGP) as described (*17*). The EITB LLGP uses a semipurified fraction of homogenized *T. solium* cysts containing 7 *T. solium* glycoprotein antigens named after the Kda molecular weights of the corresponding reactive bands (GP50, GP42, GP24, GP21, GP18, GP14, GP13). Reaction to any of these 7 glycoprotein antigens is considered positive. When applied in community settings, the EITB LLGP provides an estimate of population exposure to *T. solium* cyst antigens. A positive EITB LLGP result alone does not definitely establish active infection because antibodies can persist even after parasite clearance. The clinical significance of specific glycoprotein bands or combinations of bands in community studies has not been described. Although a highly sensitive and specific EITB is available to detect serum antibodies against adult *T. solium* intestinal infection, the unknown duration of antibody persistence after parasite clearance and the large sample size required for reasonable confidence intervals precluded our use of this assay in this study (*18*).

### Data Analysis

Data were analyzed by using Stata version 10 (StataCorp LP, College Station, TX, USA). Direct standardization was used to facilitate comparison across refugee groups, with age–sex standardized seroprevalence calculated as the weighted average of stratum-specific seroprevalence. Continuous variables were assessed by using Kruskal-Wallis for differences among groups of interest. Pearson χ^2^ and Fisher exact tests were used to compare distributions of proportions or to examine association between pairs of categoric measures. Logistic regression models were constructed for each refugee population to calculate odds ratios for seropositivity among strata (refugee camp or birth country) while controlling for age and sex. All tests are 2-sided, and significance was set at 0.05.

## Results

A total of 2,001 samples were distributed approximately equally among refugees from Burma (499 [24.9%] refugees), Laos (502 [25.1%]), Bhutan (500 [25.0%]), and Burundi (500 [25.0%]). The median age of refugees sampled was 26 years (interquartile range 20–40 years, range 15–99 years). No significant difference existed between the proportions of samples from male (984 [49.2%]) and female (1,017 [50.8%]) refugees (p = 0.30). Of the 2,001 samples, 22.5% (95% CI 20.7%–24.4%) were EITB LLGP–positive for antibodies against *T. solium* cysts.

**Figure 2:**
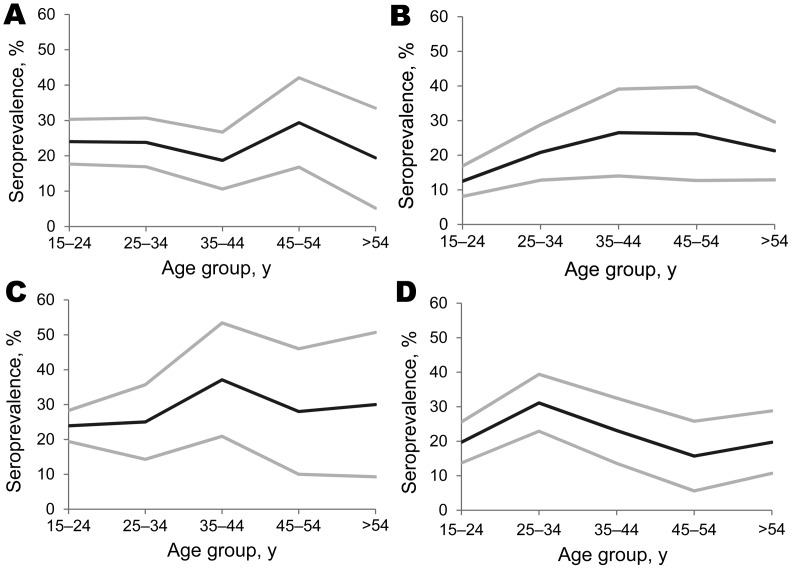
Distribution of positive results from the classic enzyme-linked immunoelectrotransfer blot for lentil-lectin purified glycoprotein for antibodies against *Taenia solium* cysticerci by age category among US-bound refugees from A) Burma (Myanmar), p = 0.65; B) Laos (Hmong), p = 0.04; C) Burundi, p = 0.56; and D) Bhutan, p = 0.12. Black lines represent seroprevalence estimates across age categories; gray lines represent upper and lower bounds of the corresponding 95% CI. Two-sided p values were determined by using likelihood ratio χ^2^.

The aggregate seroprevalence was statistically homogenous across categories of age and sex. However, within individual refugee groups, seroprevalence differed across strata of age (Figure 2) and sex (Table 1). Male refugees from Burma were 2× more likely than female refugees from Burma to be seropositive (odds ratio [OR] 2.0, 95% CI 1.3–3.1). This association between male sex and positive serologic test results was not present in the other refugee groups. The proportion of seropositive results also varied by age category in refugees from Laos (p = 0.04) and Bhutan (p = 0.12).

**Table 1:** Relationship between sex and seroprevalence of antibodies against *Taenia solium* cysts among refugees resettled in the United States

Country of origin	Male refugees			Female refugees		Odds ratio* (95% CI)	p value†
	No. positive/total no.	% (95% CI)		No. positive/total no.	% (95% CI)		
Burma (Myanmar)	78/273	28.6 (23.2–34.0)		38/226	16.8 (11.9–21.7)	2.0 (1.3–3.1)	<0.01
Laos	41/240	17.1 (12.3–21.9)		51/262	19.5 (14.7–24.3)	0.9 (0.5–1.3)	0.49
Burundi	57/234	24.4 (18.8–29.9)		72/266	27.1 (21.7–32.4)	0.9 (0.6–1.3)	0.49
Bhutan	56/237	23.6 (18.2–29.1)		58/263	22.1 (17.0–27.1)	1.1 (0.7–1.7)	0.68
Total	232/984	23.6 (20.9–26.2)		219/1,017	21.5 (19.0–24.1)	1.1 (0.9–1.4)	0.27

*Odds of positive result for enzyme-linked immunoelectrotransfer blot for lentil-lectin purified glycoprotein (EITB LLGP) testing of samples from male refugees compared with samples from female refugees. †Pearson χ^2^.

Refugees from Burundi were significantly younger than those from the other countries (p<0.01), but Burma had a higher proportion of male refugees (p = 0.04) (Table 2). The crude seroprevalence (25.8%, 95% CI 22.0–29.6) and age–sex standardized seroprevalence (27.4%, 95% CI 22.8–32.0) were highest among refugees from Burundi. Samples from Burundian refugees were collected during 2006–2007 from persons in a single camp (Kibondo, Tanzania).

**Table 2:** Crude and age–sex standardized seroprevalence of antibodies against *Taenia solium* cysts among refugees resettled in the United States

Variable	Burma, n = 499	Laos, n = 502	Bhutan, n = 500	Burundi, n = 500	p value*
Age, y, median (interquartile range)	29 (22–40)	28 (20–47)	30 (22–45)	21 (18–25)	<0.01†
Male, no. (%)	273 (54.7)	240 (47.8)	237 (47.4)	234 (46.8)	0.04
Seroprevalence, % (95% CI)					
Crude	23.2 (19.5–27.0)	18.3 (14.9–21.7)	22.8 (19.1–26.5)	25.8 (22.0–29.6)	0.04
Age–sex standardized‡	23.0 (19.1–26.8)	18.3 (14.9–21.7)	22.3 (18.5–26.0)	27.4 (22.8–32.0)	<0.01

*Pearson χ^2^ unless otherwise noted. †Kruskall-Wallis χ^2^. ‡Direct standardization method.

**Figure 3:**
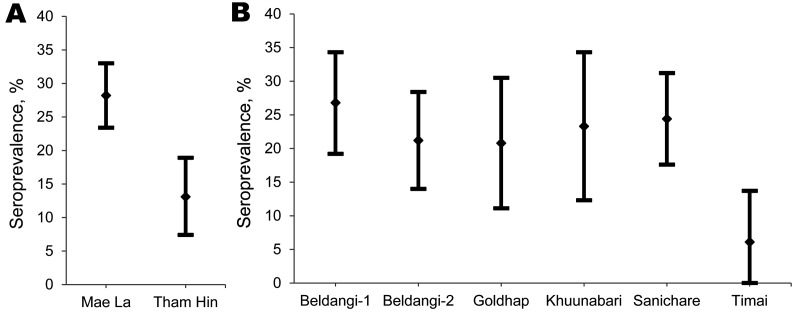
Age- and sex-adjusted seroprevalence of antibodies against *Taenia solium* cysticerci, by refugee camp among US-bound refugees from A) Burma (Myanmar) and B) Bhutan. Adjustment is by direct standardization within each refugee group. Point estimates and corresponding 95% CIs are shown.

Of the 499 samples from refugees from Burma, 459 (92.0%) were collected during 2006–2007, a period of increased resettlement; the remaining 40 were collected during 2004–2005. The refugees came from 5 refugee camps and 2 urban populations, with most samples from Mae La Camp (326 [65.3%]) and Tham Hin Camp (130 [26.1%]). The proportion of seropositive refugees was not equal between camps (p<0.01). The crude seroprevalence was significantly higher in Mae La (28.5%, 95% CI 23.6%–33.4%) than in Tham Hin (12.3%, 95% CI 6.6%–18.0%). After controlling for age and sex, we found that refugees from Mae La were >3× more likely than refugees from Tham Hin to be seropositive (OR 3.5, 95% CI 1.7–7.5) (Figure 3, panel A). The remaining 40 samples from Burmese refugees were distributed among 5 refugee sites (Nupo, Umpiem, Ban Don Yang, urban Bangkok, and urban Kuala Lumpur), but sample sizes were insufficient to calculate reliable seroprevalence estimates.

All 500 refugees from Bhutan sampled resettled during 2007–2008. The crude seroprevalence was highest in Beldangi-1 Camp (26.6%, 95% CI 18.8%–34.4%) and lowest in Timai Camp (6.3%, 95% CI 0.0–14.5) (Figure 3, panel B). After adjusting for age and sex, we found that refugees from Beldangi-1 were >5× more likely than refugees from Timai to be seropositive (OR = 5.4, 95% CI 1.2–23.9).

The 502 samples from the Hmong refugees were collected during 2004–2005, and all were from 1 refugee camp (Wat Tham Krabok) in central Thailand. This group was the only one in which refugees who provided samples were born in different countries; 260 (51.8%) were born in Thailand and 242 (48.2%) were born in Laos. The crude seroprevalence was higher for Hmong refugees born in Laos (22.7%, 95% CI 17.4%–28.0%) than for those born in Thailand (14.2%, 95% CI 10.0%–18.5%). After adjusting for age and sex, we found that Hmong refugees born in Laos were 2× more likely than those born in Thailand to be seropositive (OR 2.0, 95% CI 1.0–4.2).

Of the 451 positive samples, 247 (54.8%) were reactive to a single glycoprotein antigen band only. In all 247 instances, the single-band–positive samples reacted to GP50. The remaining 204 (45.2%) positive samples reacted to multiple bands: 51 (11.3% of the 451 positive samples) with 2 bands, 119 (26.4%) with 3 bands, and 34 (7.5%) with >4 positive bands. On 24 (5.3%) of the positive samples, an atypical band pattern was noted, in which reactivity with the lower molecular weight proteins (GP13, GP14, and GP18) were present in the absence of reactivity to higher molecular weight proteins (GP50, GP42, GP24, and GP 21). This pattern was more frequent among positive samples from Burundi (16 [12.4%], p<0.01) than from Burma (6 [5.2%]), Laos (2 [2.2%]), or Bhutan (0). The odds of an atypical reaction occurring in a sample from Burundi were 6× greater than for all of the other groups combined (OR 6.2, 95% CI 2.6–14.6). The proportional distribution of atypical reactions did not differ with respect to age (p = 0.94) or sex (p = 0.54).

## Discussion

We demonstrated that exposure to *T. solium* parasitic infection is common among refugees from Burma, Laos, Burundi, and Bhutan who resettled to the United States. All 4 populations had seroprevalence of antibodies against *T. solium* cysts comparable to or higher than the seroprevalence in well-characterized *T. solium*–endemic communities in Latin America where illness attributable to NCC is common (*4**,**5**,**19**,**20*). The widespread exposure among these groups has clinical and public health implications because these populations are resettling to the United States, where the infection is not endemic and where many clinical providers are not familiar with the disease manifestations, diagnosis, or treatment.

Epilepsy and other neurologic diseases associated with *T. solium* infection may be prevalent among certain populations of resettled refugees. We were unable to characterize the prevalence of *T. solium*–related disease because of the retrospective nature of this study and the lack of clinical data accompanying the anonymous samples. However, case reports of symptomatic NCC among resettled refugees are now being reported in the literature (*11**–**15*). Clinicians who care for migrants from countries where *T. solium* infection is endemic should have a high index of suspicion for NCC when encountering seizure disorder, chronic headache, or other neurologic deficits of unknown cause. Up-to-date information on diagnosis and treatment of NCC is available in recent reviews (*21**–**23*).

Although human cysticercosis is considered a dead end in the *T. solium* life cycle, a person with taeniasis can transmit infection to others by shedding infective eggs in feces. An adult-stage tapeworm can live for several years within the human intestine and intermittently releasing proglottids containing tens of thousands of potentially infective eggs. The infrequent reports of *Taenia* spp. infections in fecal samples of resettled refugees may not reliably indicate the true prevalence of taeniasis (*24*). Routine screening of fecal samples is done by light microscopy, which has low sensitivity (<40%) for *Taenia* spp (*25**,**26*). The number of imported *T. solium* taeniasis infections can be estimated among the populations we tested by extrapolation from other communities with similar seroprevalence in areas in which it is endemic. Multiple studies using the highly sensitive (99%) coproantigen ELISA in Latin America have shown the prevalence of *Taenia* spp. taeniasis in *T. solium*–endemic communities to be 2.0%–3.5% (*19**,**27**–**30*). These estimates include both *T. solium* and *T. saginata* tapeworms because the coproantigen ELISA used in those studies does not differentiate between these species (*26*). Approximately 87,000 refugees resettled to the United States from the 4 refugee populations we sampled during 2004–2009 (*10*). By using a conservative estimate of 1% prevalence, ≈870 refugees with *T. solium* taeniasis may have entered the United States from these populations alone.

Identifying and treating taeniasis among resettling refugees could prevent further transmission of cysticercosis in destination countries. The current approach for controlling intestinal helminthes among resettling refugees involves presumptive treatment before resettlement rather than routine fecal screening (www.cdc.gov/immigrantrefugeehealth/guidelines/overseas/intestinal-parasites-overseas.html). *Taenia* spp. tapeworms are not specifically targeted, although refugees from *Schistosoma* spp.–endemic areas in Africa may receive presumptive treatment with praziquantel, which is an effective treatment for *T. solium* taeniasis. Household screening for taeniasis when cysticercosis is diagnosed in an area to which it is not endemic is an alternate approach that can identify persons with *T. solium* intestinal tapeworm infection (*6**,**31**–**35*). Clinicians who diagnose cysticercosis should consider screening the index case-patient for taeniasis and household members for taeniasis and NCC. A combination of clinical history, laboratory analysis of feces and serum, and neuroimaging may be required.

The demonstration of widespread exposure to *T. solium* tapeworms among certain refugee populations is of concern because of the potential for severe adverse events related to presumptive treatment for intestinal helminthes. Refugees resettling to the United States from Africa and Asia receive presumptive treatment for intestinal roundworms. All refugees without contraindication receive a single dose of albendazole, and refugees originating in sub-Saharan Africa receive additional treatment for schistosomiasis with praziquantel before departure. These guidelines are consistent with program strategies of presumptive treatment in parasite-endemic areas used by the World Health Organization for soil helminth infections (www.who.int/intestinal_worms/strategy/en/) and schistosomiasis (www.who.int/schistosomiasis/strategy/en/). Both medications are used in the treatment of NCC because of their ability to penetrate the CNS and to damage *T. solium* cysts. Corticosteroids are typically administered simultaneously in treatment of NCC to control resulting inflammation and to prevent neurologic complications (*22*). The long latency between CNS infection and development of symptoms means that some persons with NCC will have occult viable brain cysts. Inadvertent damage to these occult brain cysts during presumptive treatment for intestinal helminthes could precipitate an inflammatory CNS reaction in patients for whom presumptive treatment would otherwise have been contraindicated had their infection been known. Multiple case reports describe new-onset seizures in persons with underlying NCC who receive treatment with these agents (*12**,**35**–**39*). The Food and Drug Administration recently updated label precautions for albendazole and praziquantel to inform clinicians about this potential adverse event (www.accessdata.fda.gov/drugsatfda_docs/appletter/2009/020666s005,s006ltr.pdf and www.accessdata.fda.gov/drugsatfda_docs/appletter/2010/018714s012ltr.pdf).

The current CDC refugee predeparture health guidelines advise avoiding presumptive treatment for intestinal helminthes in patients with known history of cysticercosis or previous seizure (www.cdc.gov/immigrantrefugeehealth/guidelines/refugee-guidelines.html). However, refugees from *T. solium* tapeworm–endemic regions may harbor occult viable CNS cysts that could increase their risk for severe adverse events. Refugees are observed for 1–3 days after drug administration before departure; however, enhanced surveillance with systematic data collection would help inform risk-benefit analyses of these programs. Prospective studies that monitor adverse neurologic reactions after mass treatment with albendazole and/or praziquantel in *T. solium* tapeworm–endemic areas are needed to quantify the actual risk. Clinicians should be aware of potential adverse treatment events when evaluating refugees who develop neurologic symptoms after presumptive therapy and should report suspected cases by email to CDC (RefGuidelines@cdc.gov) or telephone (1-404-498-1600) and to the Food and Drug Administration though the Adverse Events Reporting System (www.fda.gov/Drugs/GuidanceComplianceRegulatoryInformation/Surveillance/AdverseDrugEffects/ucm115894.htm).

Although we cannot confirm that active transmission is occurring within the refugee camps and/or within surrounding communities on the basis of seroprevalence alone, we do suspect that active transmission is occurring for 2 reasons: 1) the populations we sampled lived in the refugee camps for years to decades before resettlement, and 2) the antibody response to *T. solium* cysts detected on EITB LLGP has been demonstrated to be transient, with ≈40% reversion from seropositive to seronegative after 1 year in serial community studies (*40*). Although we were unable to adequately explore risk factors for positive serologic findings in this study, we did detect significant differences in the odds of exposure between refugee camps. Further investigation within the camps and surrounding communities may clarify reasons for the variation observed. Areas for further study include characterizing 1) the prevalence of epilepsy and other neurologic disease associated with NCC, 2) the prevalence of and risk factors for taeniasis, 3) the prevalence of and risk factors for porcine cysticercosis, and 4) animal husbandry practices and market structure for sale of pork. Interventions, such as screening for and treatment of taeniasis, use of corrals for raising pigs, and improved sanitation infrastructure and education, may reduce transmission among refugees and ultimately prevent disease.

This study has limitations. The EITB LLGP is known to have low sensitivity for detecting single parenchymal cysts and calcified cysts alone. On the other hand, the 100% specificity to the larval stage of *T. solium* means that false-positive reactions are unlikely. Seroprevalence estimates based on the EITB LLGP are therefore likely to underestimate the overall prevalence of exposure to *T. solium* eggs in a community. Although *T. asiatica* tapeworms are co-endemic in Southeast Asia, there is no evidence for or against potential cross-reactivity of this related species on the EITB LLGP. However, *T. asiatica* cysticercosis has not been reported among humans. We preselected our sample to include refugee populations in which we expected to find evidence of endemic *T. solium* transmission. Our seroprevalence estimates should not be generalized to all resettling refugee populations, particularly those from Middle Eastern or northern African countries, to which *T. solium* tapeworms are not thought to be endemic. Our seroprevalence estimates also may not be generalizable to the broader population in the refugees’ countries of origin. Refugee populations often include ethnic minority groups whose compilation of risk factors may not represent those of the majority population in their countries of origin. Nevertheless, our study does provide seroprevalence estimates for regions in which little to no data were previously available.

Exposure to *T. solium* parasitic infection is widespread among specific refugee groups resettled to the United States. Clinicians should suspect NCC in patients from these regions who have seizure, headache, or other unexplained neurologic manifestations and should consider screening household members for additional cases. Systematic screening and treatment for taeniasis among refugees may prevent additional cases of NCC. Further investigation is needed to characterize illness and risk factors associated with *T. solium* infection in refugee populations. Additional serologic testing of stored samples from different regions of the world with the EITB LLGP can improve understanding of the global distribution of *T. solium* infection and may highlight regions that could benefit from control or elimination interventions.

## References

[1] Scharf D. Neurocysticercosis. Two hundred thirty-eight cases from a California hospital. Arch Neurol. 1988;45:777–80. 10.1001/archneur.1988.00520310087022 3291833

[2] Shandera WX, White AC, Chen JC, Diaz P, Armstrong R. Neurocysticercosis in Houston, Texas. A report of 112 cases. Medicine. 1994;73:37–52. 10.1097/00005792-199401000-00004 8309361

[3] Ndimubanzi PC, Carabin H, Budke CM, Nguyen H, Qian Y-J, Rainwater E, A systematic review of the frequency of neurocyticercosis with a focus on people with epilepsy. PLoS Negl Trop Dis. 2010;4:e870. 10.1371/journal.pntd.0000870 21072231 PMC2970544

[4] Del Brutto OH, Santibáñez R, Idrovo L, Rodrìguez S, Díaz-Calderón E, Navas C, Epilepsy and neurocysticercosis in Atahualpa: a door-to-door survey in rural coastal Ecuador. Epilepsia. 2005;46:583–7. 10.1111/j.0013-9580.2005.36504.x 15816956

[5] Montano SM, Villaran MV, Ylquimiche L, Figueroa JJ, Rodriguez S, Bautista CT, Neurocysticercosis: association between seizures, serology, and brain CT in rural Peru. Neurology. 2005;65:229–33. 10.1212/01.wnl.0000168828.83461.09 16043791

[6] Sorvillo FJ, Waterman SH, Richards FO, Schantz PM. Cysticercosis surveillance: locally acquired and travel-related infections and detection of intestinal tapeworm carriers in Los Angeles County. Am J Trop Med Hyg. 1992;47:365–71. 1524150 10.4269/ajtmh.1992.47.365

[7] Townes JM, Hoffmann CJ, Kohn MA. Neurocysticercosis in Oregon, 1995–2000. Emerg Infect Dis. 2004;10:508–10. 15109424 10.3201/eid1003.030542 PMC3322801

[8] del la Garza Y, Graviss EA, Daver NG, Gambarin KJ, Shandera WX, Schantz PM, Epidemiology of neurocysticercosis in Houston, Texas. Am J Trop Med Hyg. 2005;73:766–70. 16222023

[9] O’Neal S, Noh J, Wilkins P, Keene W, Andersen J, Lambert W, Surveillance and screening for *Taenia solium* infection, Oregon, USA. Emerg Infect Dis. 2011;17:1030–6. 21749764 10.3201/eid1706.101397 PMC3320238

[10] US Department of Health and Human Services, Administration for Children and Families, Office of Refugee Resettlement. Refugee arrival data [cited 2011 June 9]. http://www.acf.hhs.gov/programs/orr/data/refugee_arrival_data.htm

[11] Lucey JM, McCarthy J, Burgner DP. Encysted seizures: status epilepticus in a recently resettled refugee child. Med J Aust. 2010;192:237. 20170467 10.5694/j.1326-5377.2010.tb03490.x

[12] Hewagama SS, Darby JD, Sheorey H, Daffy JR. Seizures related to praziquantel therapy in neurocysticercosis. Med J Aust. 2010;193:246–7. 20712550 10.5694/j.1326-5377.2010.tb03885.x

[13] Yeaney GA, Kolar BS, Silberstein HJ, Wang HZ. Case 163: solitary neurocysticercosis. Radiology. 2010;257:581–5. 10.1148/radiol.10090856 20959550

[14] Pluschke M, Bennett G. Orbital cysticercosis. Aust N Z J Ophthalmol. 1998;26:333–6. 10.1111/j.1442-9071.1998.tb01339.x 9843263

[15] Centers for Disease Control and Prevention. Japanese encephalitis in two children—United States, 2010. MMWR Morb Mortal Wkly Rep. 2011;60:276–8. 21389931

[16] Román G, Sotelo J, Del Brutto O, Flisser A, Dumas M, Wadia N, A proposal to declare neurocysticercosis an international reportable disease. Bull World Health Organ. 2000;78:399–406. 10812740 PMC2560715

[17] Tsang VCW, Brand JA, Boyer AE. An enzyme-linked immunoelectrotransfer blot assay and glycoprotein antigens for diagnosing human cysticercosis (*Taenia solium*). J Infect Dis. 1989;159:50–9. 10.1093/infdis/159.1.50 2909643

[18] Levine MZ, Lewis MM, Rodriguez S, Jimenez JA, Khan A, Lin S, Development of an enzyme-linked immunoelectrotransfer blot (EITB) assay using two baculovirus expressed recombinant antigens for diagnosis of *Taenia solium* taeniasis. J Parasitol. 2007;93:409–17. 10.1645/GE-938R.1 17539427

[19] García HH, Gilman RH, Gonzalez AE, Verastegui M, Rodriguez S, Gavidia C, Hyperendemic human and porcine *Taenia solium* infection in Perú. Am J Trop Med Hyg. 2003;68:268–75. 12685628

[20] Jafri HS, Torrico F, Noh JC, Bryan RT, Balderrama F, Pilcher JB, Application of the enzyme-linked immunoelectrotransfer blot to filter paper blood spots to estimate seroprevalence of cysticercosis in Bolivia. Am J Trop Med Hyg. 1998;58:313–5. 9546409 10.4269/ajtmh.1998.58.313

[21] White AC. New developments in the management of neurocysticercosis. J Infect Dis. 2009;199:1261–2. 10.1086/597758 19358667

[22] García HH, Evans CAW, Nash TE, Takayanagui OM, White AC, Botero D, Current consensus guidelines for treatment of neurocysticercosis. Clin Microbiol Rev. 2002;15:747–56. 10.1128/CMR.15.4.747-756.2002 12364377 PMC126865

[23] Garcia HH, Del Brutto OH. Neurocysticercosis: updated concepts about an old disease. Lancet Neurol. 2005;4:653–61. 10.1016/S1474-4422(05)70194-0 16168934

[24] Cartwright CP. Utility of multiple-stool-specimen ova and parasite examinations in a high-prevalence setting. J Clin Microbiol. 1999;37:2408–11. 10405376 10.1128/jcm.37.8.2408-2411.1999 PMC85240

[25] Allan JC, Craig PS. Coproantigens in taeniasis and echinococcosis. Parasitol Int. 2006;55(Suppl):S75–80. 10.1016/j.parint.2005.11.010 16337428

[26] Allan JC, Velasquez-Tohom M, Torres-Alvarez R, Yurrita P, Garcia-Noval J. Field trial of the coproantigen-based diagnosis of *Taenia solium* taeniasis by enzyme-linked immunosorbent assay. Am J Trop Med Hyg. 1996;54:352–6. 8615446 10.4269/ajtmh.1996.54.352

[27] Allan JC, Velasquez-Tohom M, Fletes C, Torres-Alvarez R, Lopez-Virula G, Yurrita P, Mass chemotherapy for intestinal *Taenia solium* infection: effect on prevalence in humans and pigs. Trans R Soc Trop Med Hyg. 1997;91:595–8. 10.1016/S0035-9203(97)90042-0 9463679

[28] Sánchez AL, Lindbäck J, Schantz PM, Sone M, Sakai H, Medina MT, A population-based, case-control study of *Taenia solium* taeniasis and cysticercosis. Ann Trop Med Parasitol. 1999;93:247–58. 10.1080/00034989958500 10562826

[29] Sarti E, Schantz P, Avila G, Ambrosio J, Medina-Santillan R, Flisser A. Mass treatment against human taeniasis for the control of cysticercosis: a population-based intervention study. Trans R Soc Trop Med Hyg. 2000;94:85–9. 10.1016/S0035-9203(00)90451-6 10748908

[30] Allan JC, Velasquez-Tohom M, Garcia-Noval J, Torres-Alvarez R, Yurrita P, Fletes C, Epidemiology of intestinal taeniasis in four, rural, Guatemalan communities. Ann Trop Med Parasitol. 1996;90:157–65. 8762405 10.1080/00034983.1996.11813039

[31] Kruskal BA, Moths L, Teele DW. Neurocysticercosis in a child with no history of travel outside the continental United States. Clin Infect Dis. 1993;16:290–2. 10.1093/clind/16.2.290 8443310

[32] Asnis D, Kazakov J, Toronjadze T, Bern C, Garcia HH, McAuliffe I, Neurocysticercosis in the infant of a pregnant mother with a tapeworm. Am J Trop Med Hyg. 2009;81:449–51. 19706913

[33] Tasker WG, Plotkin SA. Cerebral cysticercosis. Pediatrics. 1979;63:761–3. 440898

[34] Schantz PM, Moore AC, Muñoz JL, Hartman BJ, Schaefer JA, Aron AM, Neurocysticercosis in an Orthodox Jewish community in New York City. N Engl J Med. 1992;327:692–5. 10.1056/NEJM199209033271004 1495521

[35] Garcia HH, Gonzalez I, Mija L. Neurocysticercosis uncovered by single-dose albendazole. N Engl J Med. 2007;356:1277–8. 10.1056/NEJMc062891 17377173

[36] Torres JR, Noya O, de Noya BA, Mondolfi A. Seizures and praziquantel. A case report. Rev Inst Med Trop Sao Paulo. 1988;30:433–6. 10.1590/S0036-46651988000600008 3252438

[37] Torres JR. Use of praziquantel in populations at risk of neurocysticercosis. Rev Inst Med Trop Sao Paulo. 1989;31:290. 10.1590/S0036-46651989000400014 2626648

[38] Lillie P, McGann H. Empiric albendazole therapy and new onset seizures—a cautionary note. J Infect. 2010;60:403–4. Author reply 404–5. 10.1016/j.jinf.2010.02.004 20153773

[39] Flisser A, Madrazo I, Plancarte A, Schantz P, Allan J, Craig P, Neurological symptoms in occult neurocysticercosis after single taeniacidal dose of praziquantel. Lancet. 1993;342:748. 10.1016/0140-6736(93)91743-6 8103859

[40] Garcia HH, Gonzalez AE, Gilman RH, Palacios LG, Jimenez I, Rodriguez S, Short report: transient antibody response in *Taenia solium* infection in field conditions—a major contributor to high seroprevalence. Am J Trop Med Hyg. 2001;65:31–2. 11504404 10.4269/ajtmh.2001.65.31

